# Supplementary figures and images for: New β-Propellers Are Continuously Amplified From Single Blades in all Major Lineages of the β-Propeller Superfamily
Source: Front Mol Biosci. 2022 Jun 9;9:895496. doi: 10.3389/fmolb.2022.895496 (PMC9218822; doi:10.3389/fmolb.2022.895496)

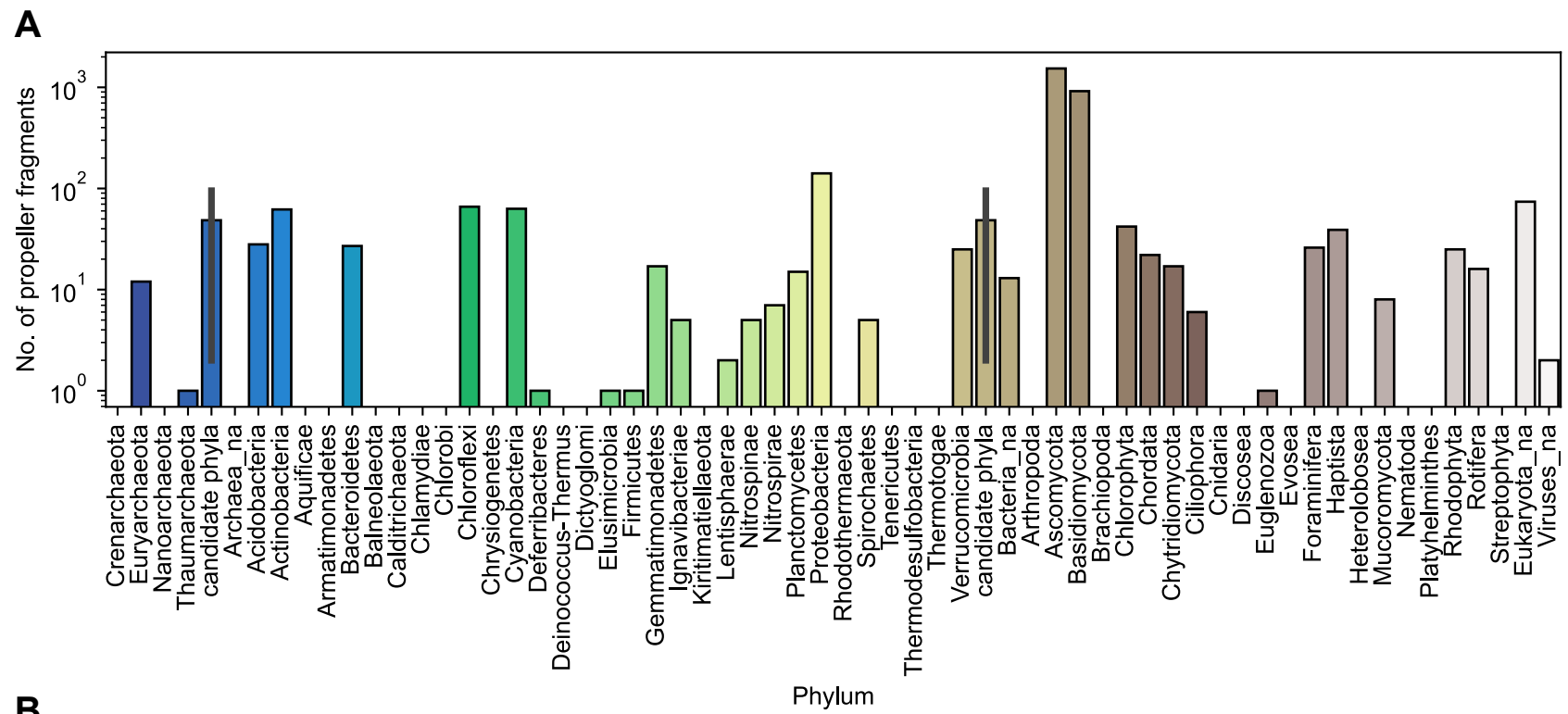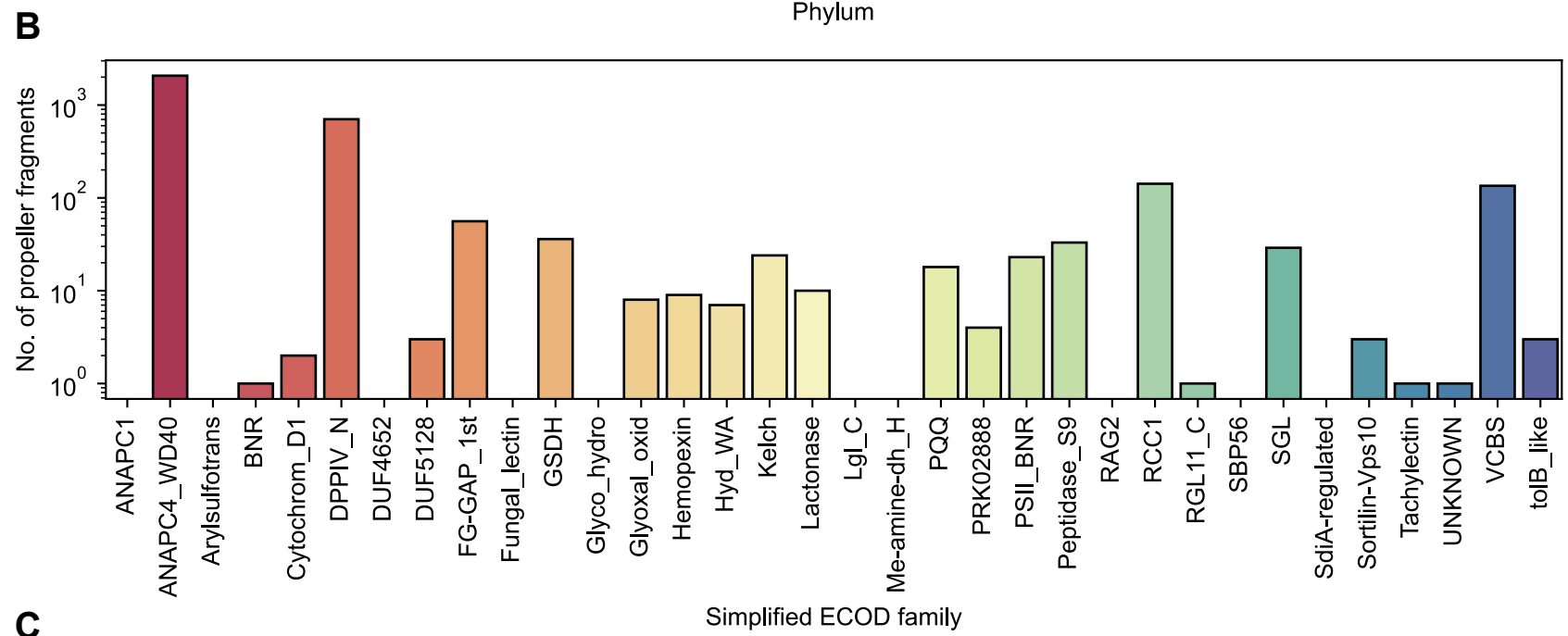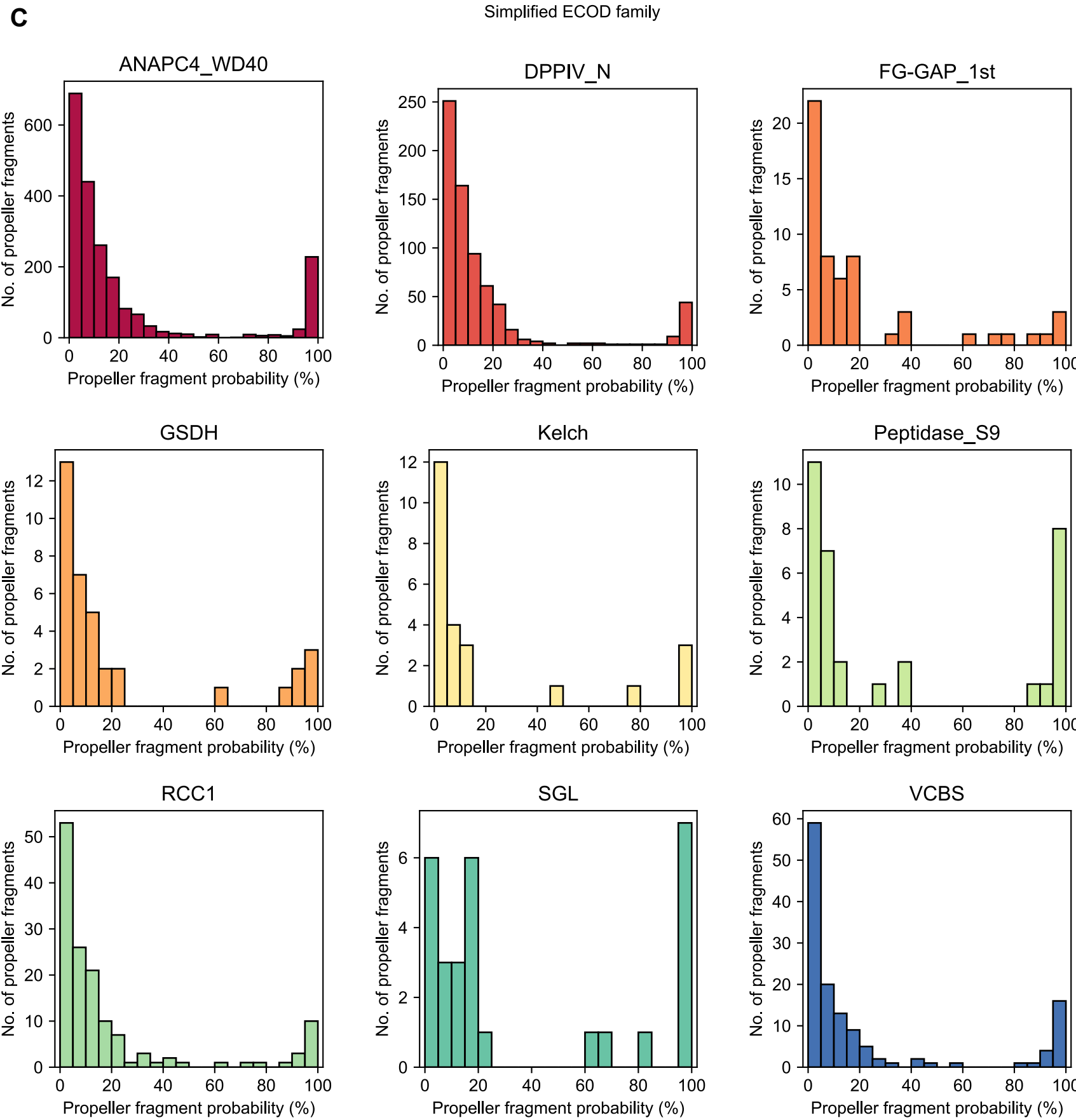

Supplement: Supplementary file 1 [file DataSheet7.PDF]

**A**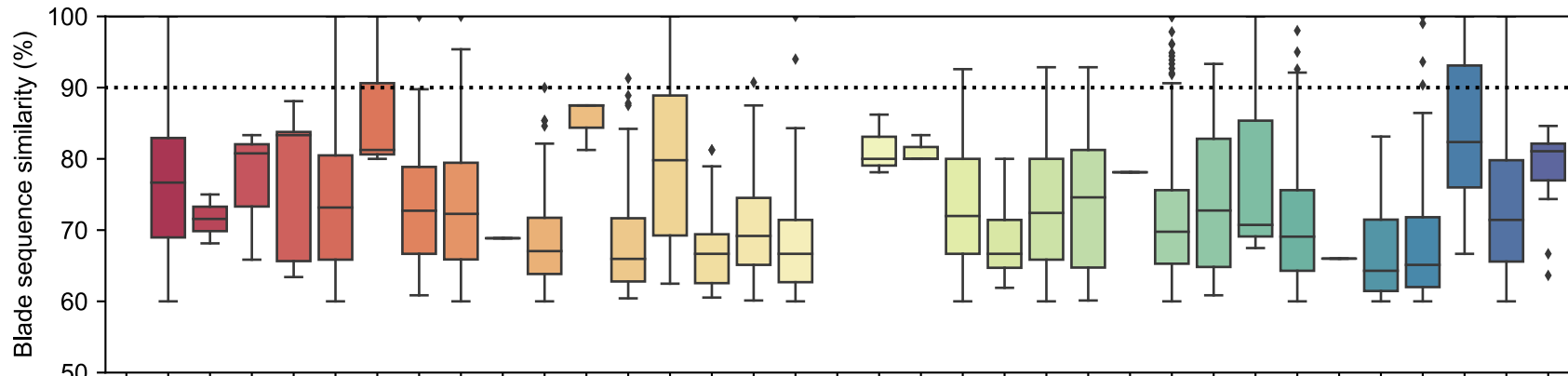**B**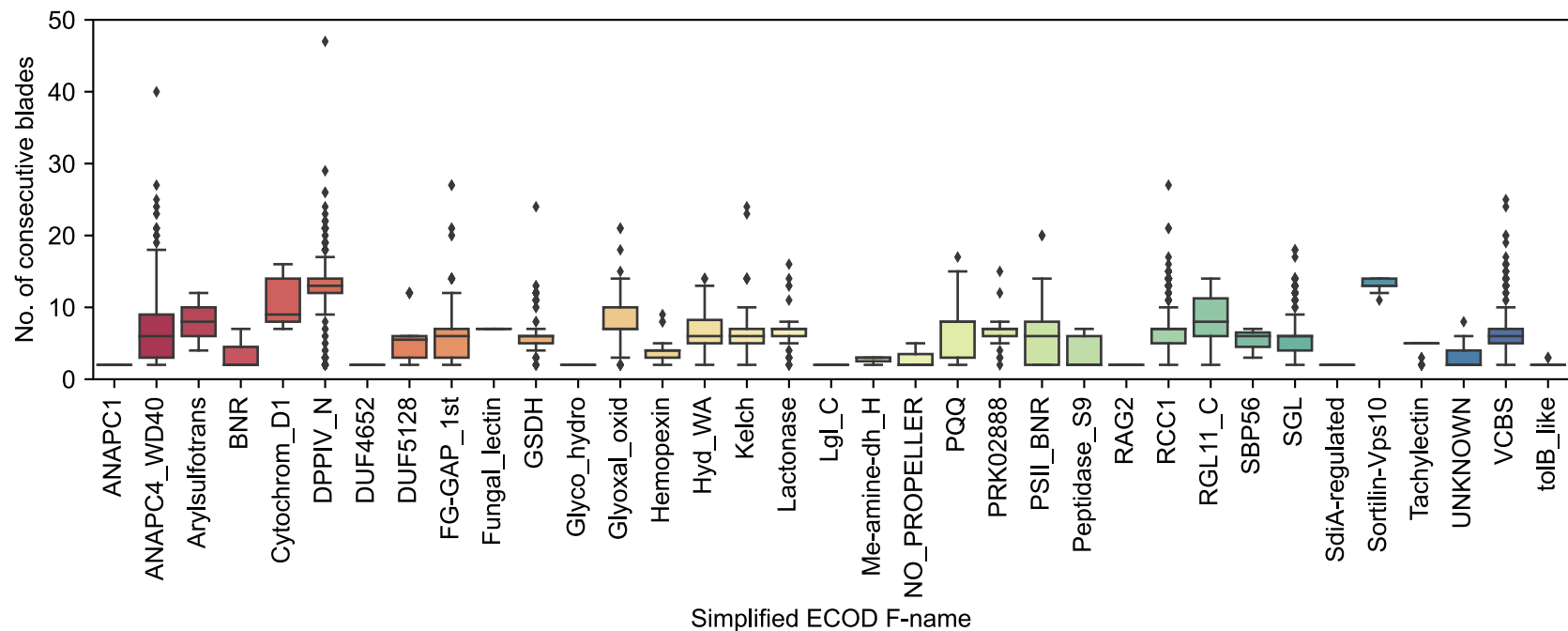

Supplement: Supplementary file 2 [file DataSheet2.PDF]

ANAPC4\_WD40

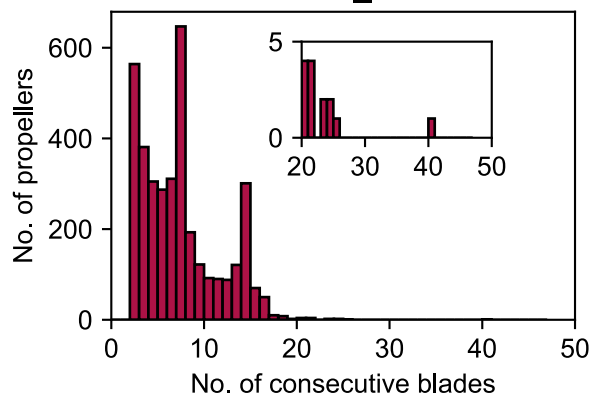

DPPIV\_N

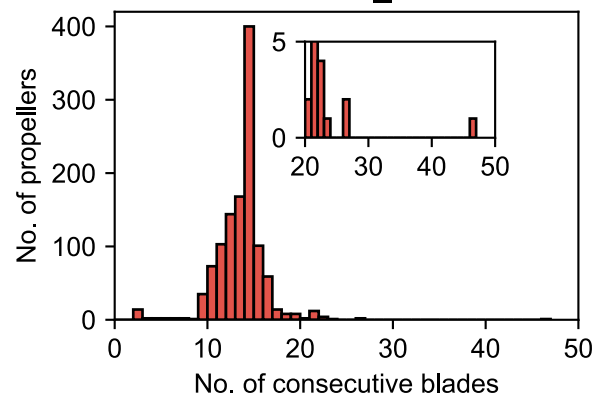

FG-GAP\_1st

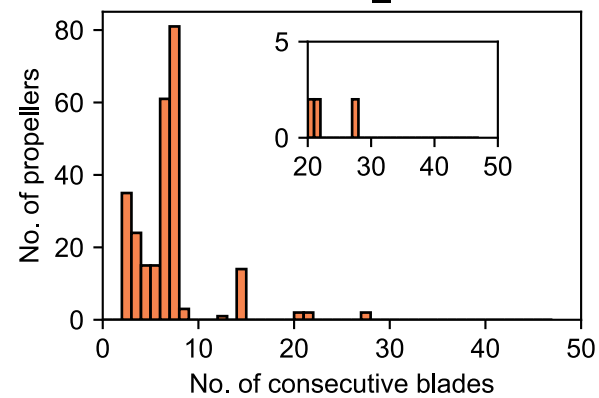

GSDH

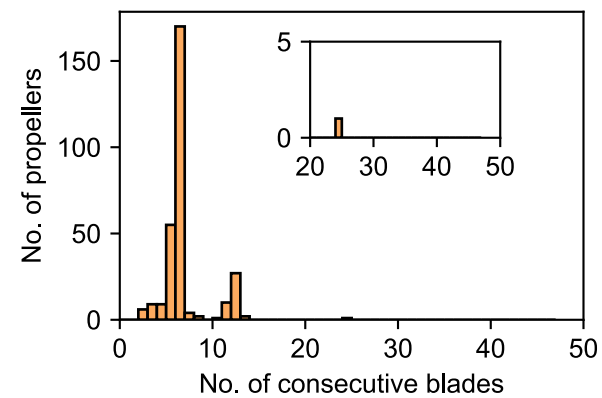

PQQ

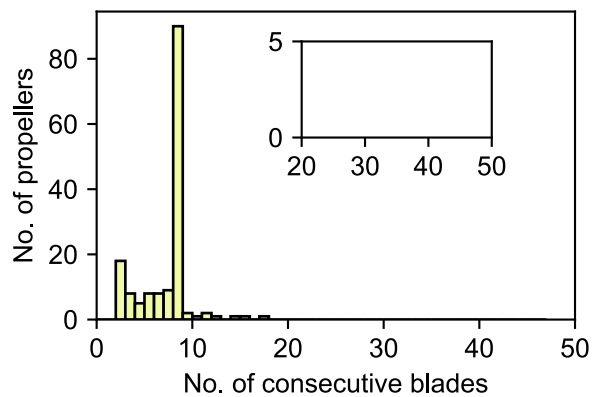

RCC1

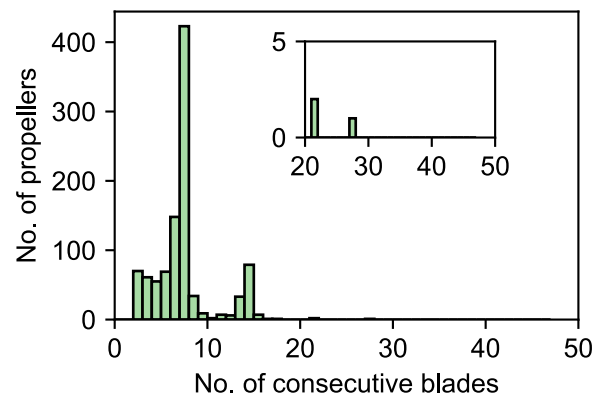

SGL

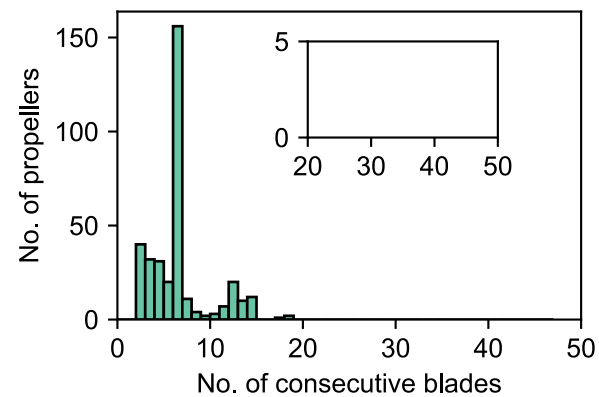

VCBS

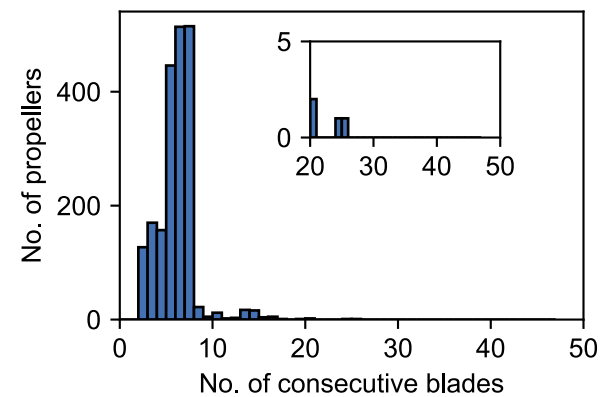

Supplement: Supplementary file 7 [file DataSheet5.PDF]
