# Supplementary material for: New β-Propellers Are Continuously Amplified From Single Blades in all Major Lineages of the β-Propeller Superfamily
Source: Front Mol Biosci. 2022 Jun 9;9:895496. doi: 10.3389/fmolb.2022.895496 (PMC9218822; doi:10.3389/fmolb.2022.895496)
Supplement: Supplementary file 3 [file DataSheet4.PDF]

A QFS50082.1 chitinase GXM_07576 [Nostoc sphaeroides CCNUC1]
3 blades

```
                                          MYRTKKLATKN-FSLLQ-AWINFG
KYPRLVADVNDDSQTDIVGFGYDNMFVSFEPSNRSFGEAFVAKNDDFTFNKGAWASLD
CYPRQLAKLNGNSRVDIVDFGYDNVFMSFGKSNGTFDEVFVAKNYNFTNRKDYCNSFD
QKPRQLGDVNGDGKADILSFAQDGTYVALA
NNQTTPPPMHNQYVVAGYLPSWGISNTTNPETIPVNKLTHLFYAFADVDTQGNVKLSQDGQDGDINLLKC
LKAQNPKLKILVSIGGAGENDFSSAASTTQSRIFFAQSAINFMKSNGFDGIDINWEFPKKEENTNYTQLL
GEIRQQLNNASTTDGKKYLLTTALSASPYQLSPSDYADAPYDLNSTVLKTTSEYVDFINVMTYDYHGSWE
NTTNHQAALYKSSSDQSYNSDKLNADWSVKKYLSAGVEAKDIVLGVPLYSPSWAGVKPGSDNDGLFQSAT
PVNDPLLYRDIHDRVGTNGYQYYLDDSAKVPYIYNSQKQEFSTYEDKQSVLEKVNYVEQQGLGGIFFWQL
LGDLPINHSDSLVNVAANLLYNLL
```

B MCC5615739.1 FG-GAP-like repeat-containing protein [Nostoc sp. CHAB 5836]
85% identical to GXM_07576 above; 6 blades

```
                                          MYGTKKLALNK-FSLLQ-KWTNFG
KFPRQVADVNGDGRTDIVGFGCDAVYVSLGKNNGTFSHAFIARKDNFTAKKGGWTSFN
KYPRQLADVNGDGRADIVGFATNAVYVSLGQSNGTFGQAFTGINDNFTVNKGRWTSFD
KYPRQLADVNGDGRADIVGFGYDNVSVSLGQSNGTFGSTSVAKNDDFTVNKGGWTSFD
KYPRQLADVNGDGRADIVGFGYDNVSVALGQSNGTFGSTSVAKNDDFTVNKGGWNSFD
KYPRQLADVNGDGRADIVGFGYDNVSVALGQSNGTFGSTSVAKNDDFTVNKGGWNSFD
QKPRQLGDVNGDGKADIIGFAQDGTYVALA
NNQTTSPPMNNQYIVAGYLPSWGISSTTNPATIPVNKLTHLFYAFADVDTQGNVKLSEDGQDGDINVLKS
LQAQNPKLKILVSIGGAGENDFSSAASTAQSRIFFAQSAINFMKSNGFDGIDINWEFPKKEENSNYTQLL
GELRQQLNNASTTDGKKYLLTTALSASPYQLSPSDYADAPYDLNSTVLKTTSEYVDFINVMTYDYHGSWE
NTTNHQAPLYKSSSDQSYNSDKLNADWSVKKYLSAGVEAKDIVLGVPLYSPTWTGVKPGSNNDGLFQSAT
PVNDPLLYRDLHDQVGTNGYKYYWDDSAKVPYIYNSEKQEFSTYEDKQSVLGKVNYVEEQGLGGVFFWQL
LGDLPITHSDSLVNVAAANLL
```

C AUB42408.1 chitinase COO91_08536 [Nostoc flagelliforme CCNUN1]
83% identical to GXM_07576 above; 3 blades with large internal deletion (strands 3 and 4) in blade 1

```
                                          MYRTKKLGIKK-FSFLQ-KWINIG
KDPHQVANVNGDARTNIVGFGYNAI-----------------NDNFTFNKGEWTSFD
KYPRQLADVNGDSRADIVGFGYDNVFVSLGQSNGTFGQAFVAQNDDFTVNQSGWNSFE
QKPRQLGDVNGDAKADIIGFAQDGTYVALA
NNQTTPPPINNQYVVAGYLPSWGINNTTNTATIPVNKLTHLFYAFADVDTEGNVKLSQDGEDGDINILKS
LKDQNPKLKILISIGGAGENDFSSAASTAQSRIFFAQSAINFMKSNGFDGIDINWEFPKKEENSNYTQLL
GELRQQLNNASTTDGNKYLLTTALSASPYQLSPSDYADAPYDLNSTVLKSTSEYVDFINVMTYDYHGSWE
NTTNHQAALYKSSTDQSYNSDKLNADWSVKKYLSAGVEAKDIVLGVPLYSPTWAGVKPGSNNDGLFQSAT
LGNDPLLYKDIHDQVGTNGYQYYWDDSAKVPYIYNSQKQEFSTYEDKQSVLGKVNYVEEQGLGGIFFWQL
LGDLPITHPDSLVNVAAGNLF
```

D GBG18942.1 VCBS repeat-containing protein NIES4072_26070 [Nostoc commune NIES-4072]
84% identical to GXM_07576 above, 100% identical to NIES4070_00790 below; 4 blades

```
                                          MYGIKKLAIKK-FSLLQ-KWTNFW
KFTRQVADVNGDGRTDIVAFGCDAVYVSLGQSNGTFGQAFTGITDSFTINQGEWTSFD
KYPRQLADVNGDGRADIVGFGYDNVFVSLGQSNGTFGPTSVAKNDDFTVNKGGWTSFD
KYPRQLADVNGDGRADIVGFGYDNVFVSLGQSNGTFGPTSVAKNDDFTVNKGDRNNFD
HKPRQLGDVNGDGKADIIGFAQDGTYVALA
NNQTTPPPMNNQYVVAGYLPSWGISSTTNPATIPVNKLTHLFYAFADVDTEGNVKLSPDGQDGDINVLKS
LKAQNPKLKILVSIGGAGENDFSSAASTAQSRIFFAQSAINFMKNNGFDGIDIDWEFPKKEENSNYIQLL
SELRQELNNASTTDGNKYLLTTALSGSPYQLSPSDYADAPYDLNSTVLKTTSEYVDFINLMTYDYHGSWE
NTTNHQAALYKSSSDQSYNSDKLNADWSVKKYLSAGVEAKDIVLGVPLYSPTWAGVKAGSNNDGLFQSAT
SANDPLLYKDIHAQVGTDGYQYNWDDSAKVPYIYNSQKQEFSTYEDKRSVLEKVNYVEQQGLGGIFFWQL
IGDLPITHSDSLVNVAAANLL
```

E BBD63737.1 VCBS repeat-containing protein NIES4070_00790 [Nostoc commune HK-02]
84% identical to GXM_07576 above, 100% identical to NIES4070_00790 above; 6 blades

```
                                          MYGIKKLAIKK-FSLLQ-KWTNFW
KFTRQVADVNGDGRTDIVAFGCDAVYVSLGQSNGTFGQAFTGITDSFTINQGEWTSFD
KYPRQLADVNGDGRADIVGFGYDNVFVSLGQSNGTFGPTSVAKNDDFTVNKGGWTSFD
KYPRQLADVNGDGRADIVGFGYDNVFVSLGQSNGTFGPTSVAKNDDFTVNKGGWTSFD
KYPRQLADVNGDGRADIVGFGYDNVFVSLGQSNGTFGPTSVAKNDDFTVNKGGWTSFD
KYPRQLADVNGDGRADIVGFGYDNVFVSLGQSNGTFGPTSVAKNDDFTVNKGDRNNFD
HKPRQLGDVNGDGKADIIGFAQDGTYVALA
NNQTTPPPMNNQYVVAGYLPSWGISSTTNPATIPVNKLTHLFYAFADVDTEGNVKLSPDGQDGDINVLKS
LKAQNPKLKILVSIGGAGENDFSSAASTAQSRIFFAQSAINFMKNNGFDGIDIDWEFPKKEENSNYIQLL
SELRQELNNASTTDGNKYLLTTALSGSPYQLSPSDYADAPYDLNSTVLKTTSEYVDFINLMTYDYHGSWE
NTTNHQAALYKSSSDQSYNSDKLNADWSVKKYLSAGVEAKDIVLGVPLYSPTWAGVKAGSNNDGLFQSAT
SANDPLLYKDIHAQVGTDGYQYNWDDSAKVPYIYNSQKQEFSTYEDKRSVLEKVNYVEQQGLGGIFFWQL
IGDLPITHSDSLVNVAAANLL
```

F KST69896.1 hypothetical protein BC008_05515 [Mastigocoleus testarum BC008]
100% identical to its paralog BC008_06320; 6 blades

```
                                          MTTFGVKKIATNN-FGHSQG-WSSFD
KYPRQIADVNGDGRDDLIAFGYDNVVVSLGESNGTFGPAFVANNDGFTVSKGDWSSFD
KYPRQVADVNGDGRADIIGFGYDKVLVSLGQSNGTFGQALIADNDGFTVSKGDWSSFD
KYPRQVADVNGDGRADIIGFGYDKVLVSLGQSNGTFGQALIADSDGFTVSKGDWSSFD
KYPRQVADVNGDGRADIIGFGYDKVLVSLGQSNGNFGQALLAKNDDFTVSKGNWSNFD
LYPRQVADVNGDGRADIVGFGPDNVQISLGQSDGTFGATTVAKNDDFTVNKGGWNSFD
TKPRQLGDVNGDGRADIVGFDQDGTYVALA
DDNNTTQPGNNERIVGGYLPSWEINGNTDPASIPGDKLTHLFYAFVDVDAQGNIKLNQDTGLDGDIDALK
SIKAQNPDLKILVSIGGAGDPDFSPTASNPQSRANFVNSAVQFMRNNGFDGIDIDWEFPKKEENDNYLKL
LGDLRQEVNKVSLTDGKDYQLTTALSASPYQLSPSDYGDSPYDLNPAVLKQTSEYVDFINVMSYDYHGPW
EQKTNHQAALYKNSNDNSYNSDKLNVSWGIQEYLNAGVDAKDIVLGVPLYSYSWTGVNPGANNDGLLQSG
TPVPGENAILYKDLYDKIDTNGYERYWDDSAQVPYVYNSQTQEFSTYEDKQSVLGKIDYLEQQELGGMFF
WHLGGDLPINNPDSLVNTAASKLMV
```

G KST70053.1 hypothetical protein BC008_06320 [Mastigocoleus testarum BC008]
9 blades

```
                                          MTTFGVKKIATNN-FGHSQG-WSSFD
KYPRQIADVNGDGRDDLIAFGYDNVVVSLGESNGTFGPAFVANNDGFTVSKGDWSSFD
KYPRQVADVNGDGRADIIGFGYDKVLVSLGQSNGTFGQALIADNDGFTVSKGDWSSFD
KYPRQVADVNGDGRADIIGFGYDKVLVSLGQSNGTFGQALIADNDGFTVSKGDWSSFD
KYPRQVADVNGDGRADIIGFGYDKVLVSLGQSNGTFGQALIADSDGFTVSKGDWSSFD
KYPRQVADVNGDGRADIIGFGYDKVLVSLGQSNGTFGQALIADNDGFTVSKGDWSSFD
KYPRQVADVNGDGRADIIGFGYDKVLVSLGQSNGTFGQALIADSDGFTVSKGDWSSFD
KYPRQVADVNGDGRADIIGFGYDKVLVSLGQSNGNFGQALLAKNDDFTVSKGNWSNFD
LYPRQVADVNGDGRADIVGFGPDNVQISLGQSDGTFGATTVAKNDDFTVNKGGWNSFD
TKPRQLGDVNGDGRADIVGFDQDGTYVALA
DDNNTTQPGNNERIVGGYLPSWEINGNTDPASIPGDKLTHLFYAFVDVDAQGNIKLNQDTGLDGDIDALK
SIKAQNPDLKILVSIGGAGDPDFSPTASNPQSRANFVNSAVQFMRNNGFDGIDIDWEFPKKEENDNYLKL
LGDLRQEVNKVSLTDGKDYQLTTALSASPYQLSPSDYGDSPYDLNPAVLKQTSEYVDFINVMSYDYHGPW
EQKTNHQAALYKNSNDNSYNSDKLNVSWGIQEYLNAGVDAKDIVLGVPLYSYSWTGVNPGANNDGLLQSG
TPVPGENAILYKDLYDKIDTNGYERYWDDSAQVPYVYNSQTQEFSTYEDKQSVLGKIDYLEQQELGGMFF
WHLGGDLPINNPDSLVNTAASKLMV
```
