# Supplementary material for: New β-Propellers Are Continuously Amplified From Single Blades in all Major Lineages of the β-Propeller Superfamily
Source: Front Mol Biosci. 2022 Jun 9;9:895496. doi: 10.3389/fmolb.2022.895496 (PMC9218822; doi:10.3389/fmolb.2022.895496)
Supplement: Supplementary file 5 [file DataSheet3.PDF]

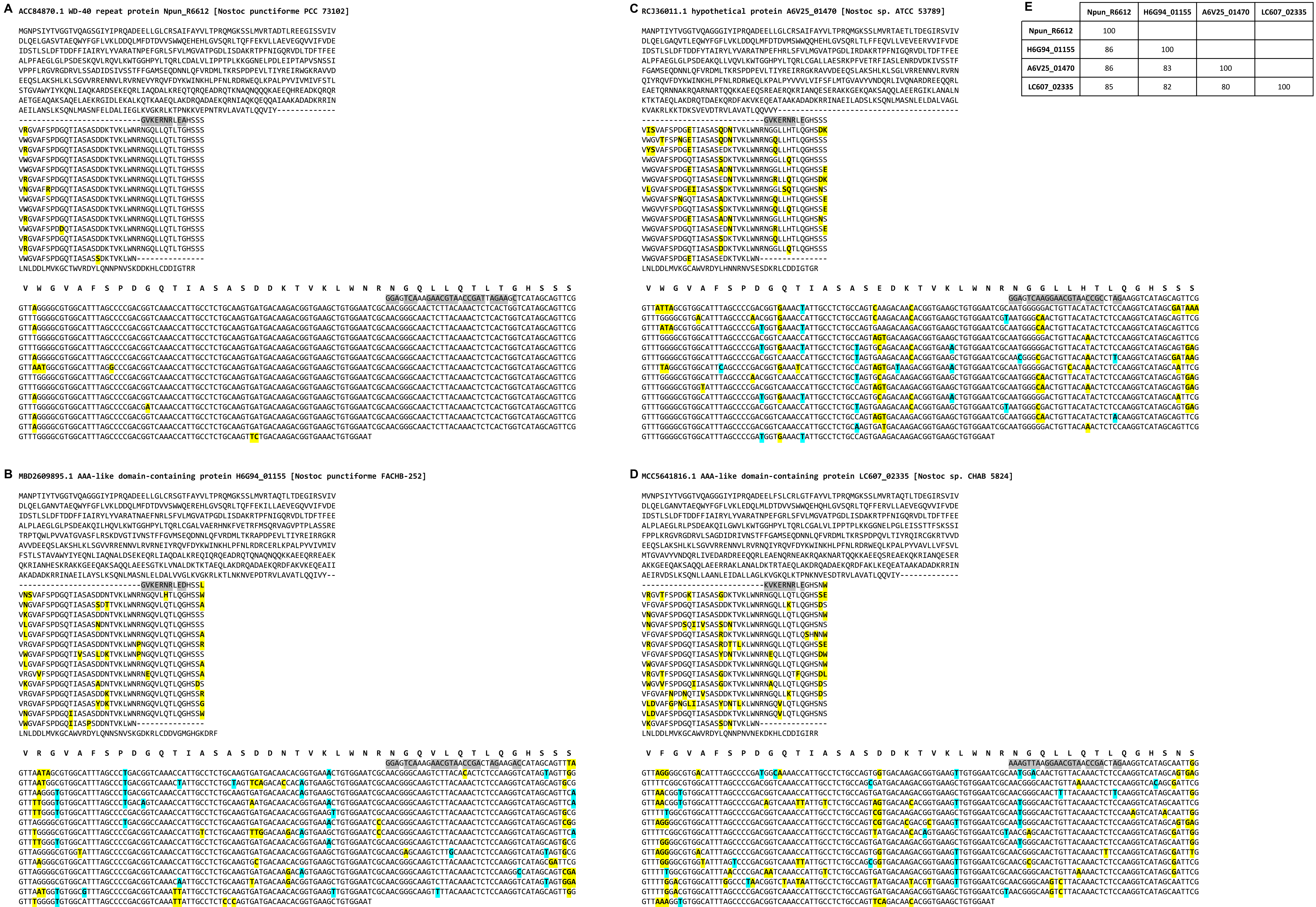
A
ACC84870.1 WD-40 repeat protein Npun_R6612 [Nostoc punctiforme PCC 73102]
B
MBD2609895.1 AAA-like domain-containing protein H6G94_01155 [Nostoc punctiforme FACHB-252]
C
RCJ36011.1 hypothetical protein A6V25_01470 [Nostoc sp. ATCC 53789]
D
MCC5641816.1 AAA-like domain-containing protein LC607_02335 [Nostoc sp. CHAB 5824]
E
	Npun_R6612	H6G94_01155	A6V25_01470	LC607_02335
Npun_R6612	100			
H6G94_01155	86	100		
A6V25_01470	86	83	100	
LC607_02335	85	82	80	100
