# Supplementary material for: New β-Propellers Are Continuously Amplified From Single Blades in all Major Lineages of the β-Propeller Superfamily
Source: Front Mol Biosci. 2022 Jun 9;9:895496. doi: 10.3389/fmolb.2022.895496 (PMC9218822; doi:10.3389/fmolb.2022.895496)
Supplement: Supplementary file 6 [file DataSheet1.pdf]

SUPPLEMETARY

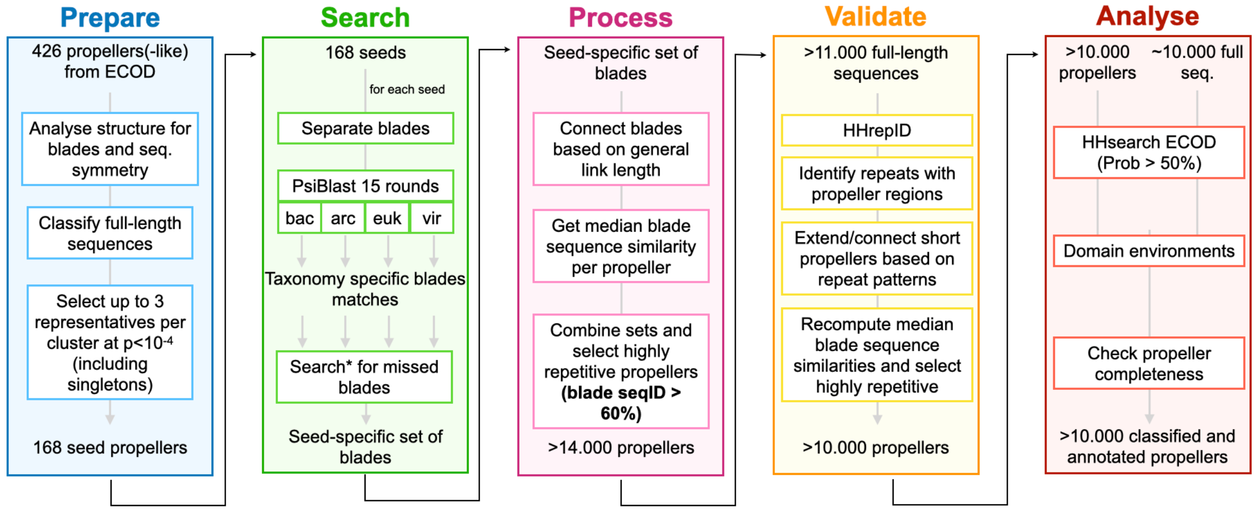

Figure S1. General workflow for the identification and annotation of protein sequences containing highly symmetric β-propellers. A detailed description of the procedure is given in the “Material and Methods” section.

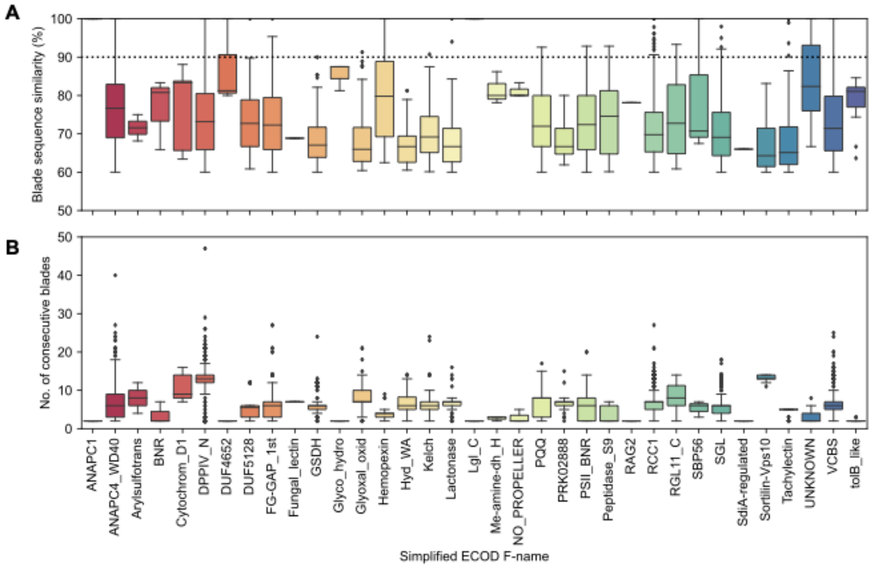

Figure S2. Boxplot of (a) the median sequence identity, and (b) the absolute number of consecutive highly similar blades for different β-propeller families.

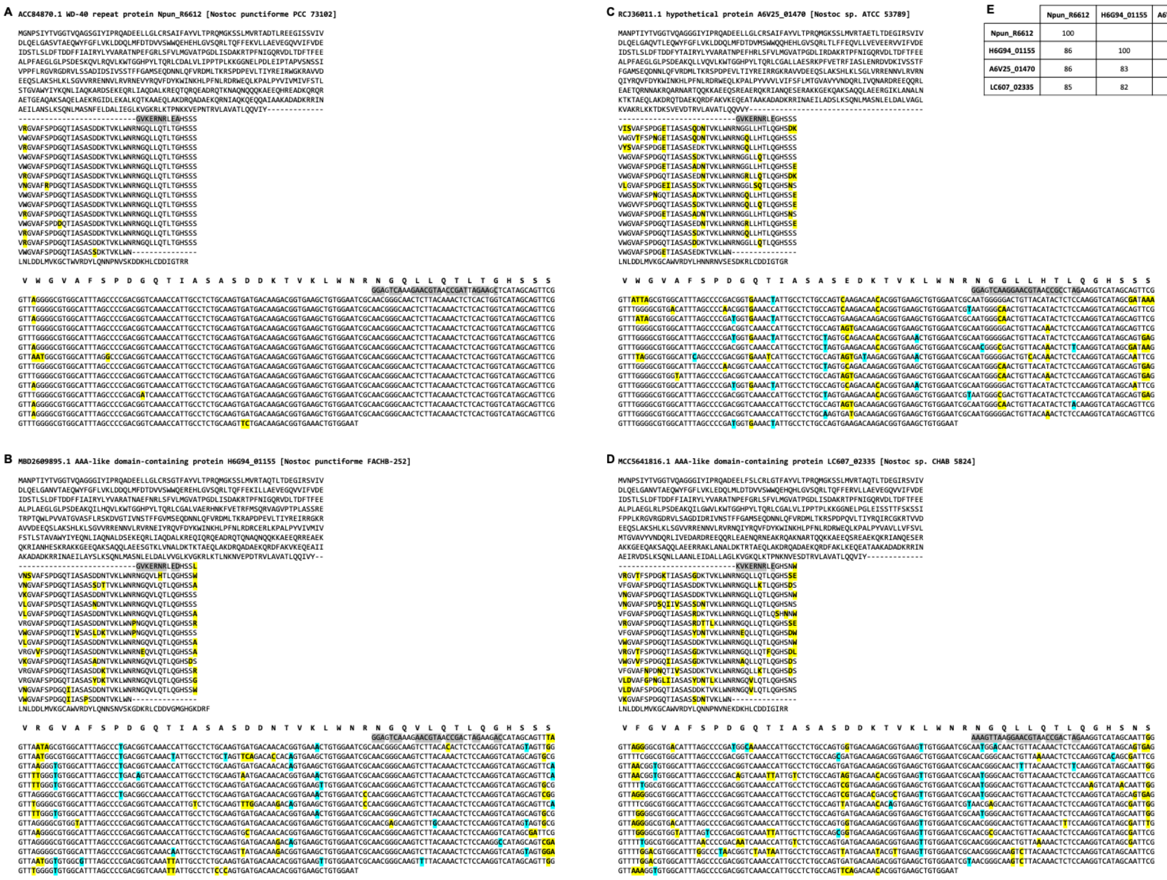

E

	Npun_R6612	H6G94_01155	A6V25_01470	LC607_02335
**Npun_R6612**	100			
**H6G94_01155**	86	100		
**A6V25_01470**	86	83	100	
**LC607_02335**	85	82	80	100

Figure S3. The four WRAP domain homologs in figure 2 and their pairwise sequence identities. (a-d) For each sequence, we show the alignment of the 14 blades with mutations highlighted in yellow and the corresponding nucleotide sequence with highlights as described in figure 2. The majority rule consensus of the β-propeller blades is shown above the nucleotide sequences. (e) Table of pairwise sequence identities.

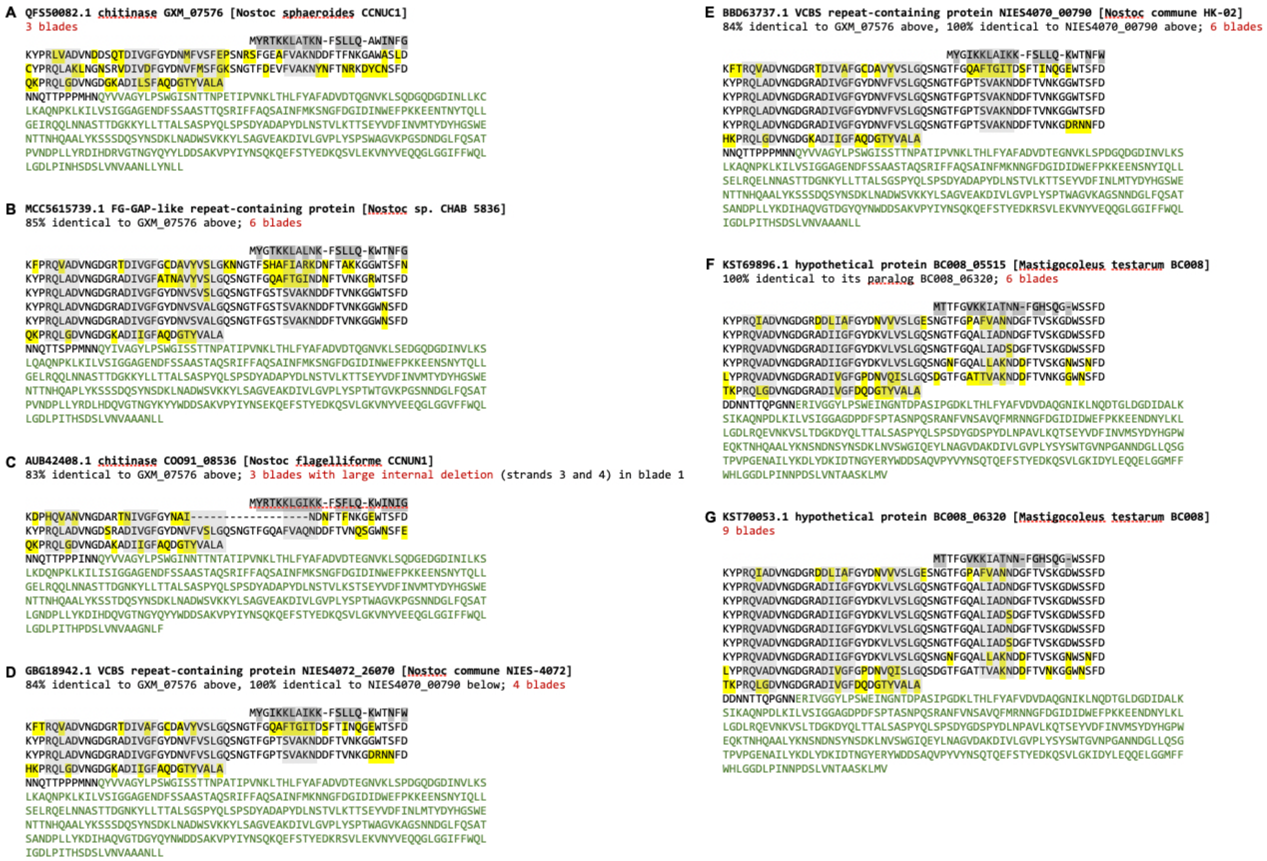

Figure S4. A family of cyanobacterial chitinases whose N-terminal propeller domain shows more recent local, protein-specific amplifications of an internal blade, nested within the older global amplification which gave rise to the domain. The local amplifications proceeded to different extent in cases where they occurred, leading from a basal 3-bladed structure (A,C) to forms with 4 (D), 6 (B,E,F), and 9 (G) blades. In all sequences, deviations from the internal consensus of the propeller are highlighted in yellow and the chitinase domains are colored green. As in Figs. 2 and S3, the sequence of the first β-strand of the structure is different from that of the equivalent strand in the amplified region and highly conserved between homologs; it is highlighted in dark grey. The strands are highlighted in light grey. It is striking that, within this group of proteins, we can observe twice cases where otherwise 100% sequence identical proteins have different blade numbers (NIES4072_26070 vs. NIES4070_00790 and BC008_05515 vs. BC008_06320). It seems likely that all proteins in this family form 6-bladed propellers, the 3-bladed sequences by dimerization, the 4-bladed one by trimerization (3x4 = 2x6), the 6-bladed ones as monomers, and the 9-bladed one by dimerization (2x9 = 3x6). In the process they would undergo metamorphic asymmetries as needed, akin to the tachylectin-2 constructs of Yadid and Tawfik (2007, 2011), but AlphaFold models do not show this for the 4-bladed and 9-bladed forms, presumably because of the difficulty of generating metamorphic asymmetries in sequence-identical subunits.

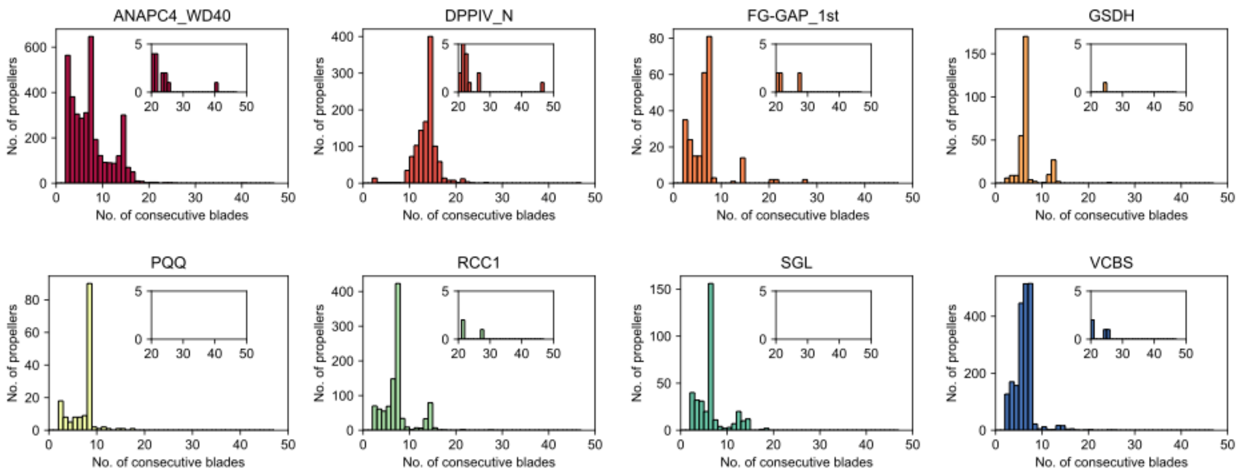

Figure S5. Histograms of the absolute number of consecutive highly identical blades for the globally highly repetitive β-propeller from the 8 most frequent families in the dataset.

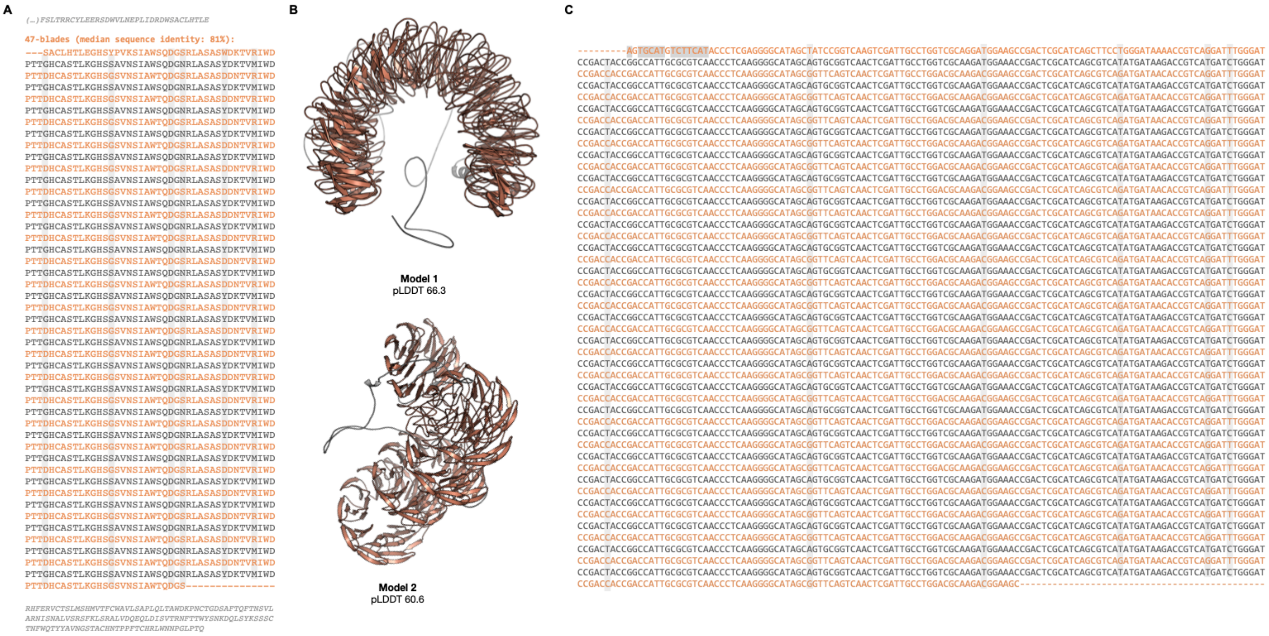

Figure S6. The 47-bladed globally highly repetitive β-propeller from *Penicillium camemberti* hypothetical NACHT nucleoside triphosphatase (CRL31138.1). (a) Annotated β-propeller sequence, highlighting the 2-step repeat pattern, plus the flanking non-propeller sequences. (b) The two best three-dimensional models of the β-propeller and immediate flanking regions predicted with AlphaFold v2.1.2 (Jumper *et al.*, 2021). (s) The nucleotide sequence coding for the highly repetitive β-propeller region highlighting the same 2-step, intercalated, repeat pattern. In (a) and (b), point mutations are highlighted in grey.

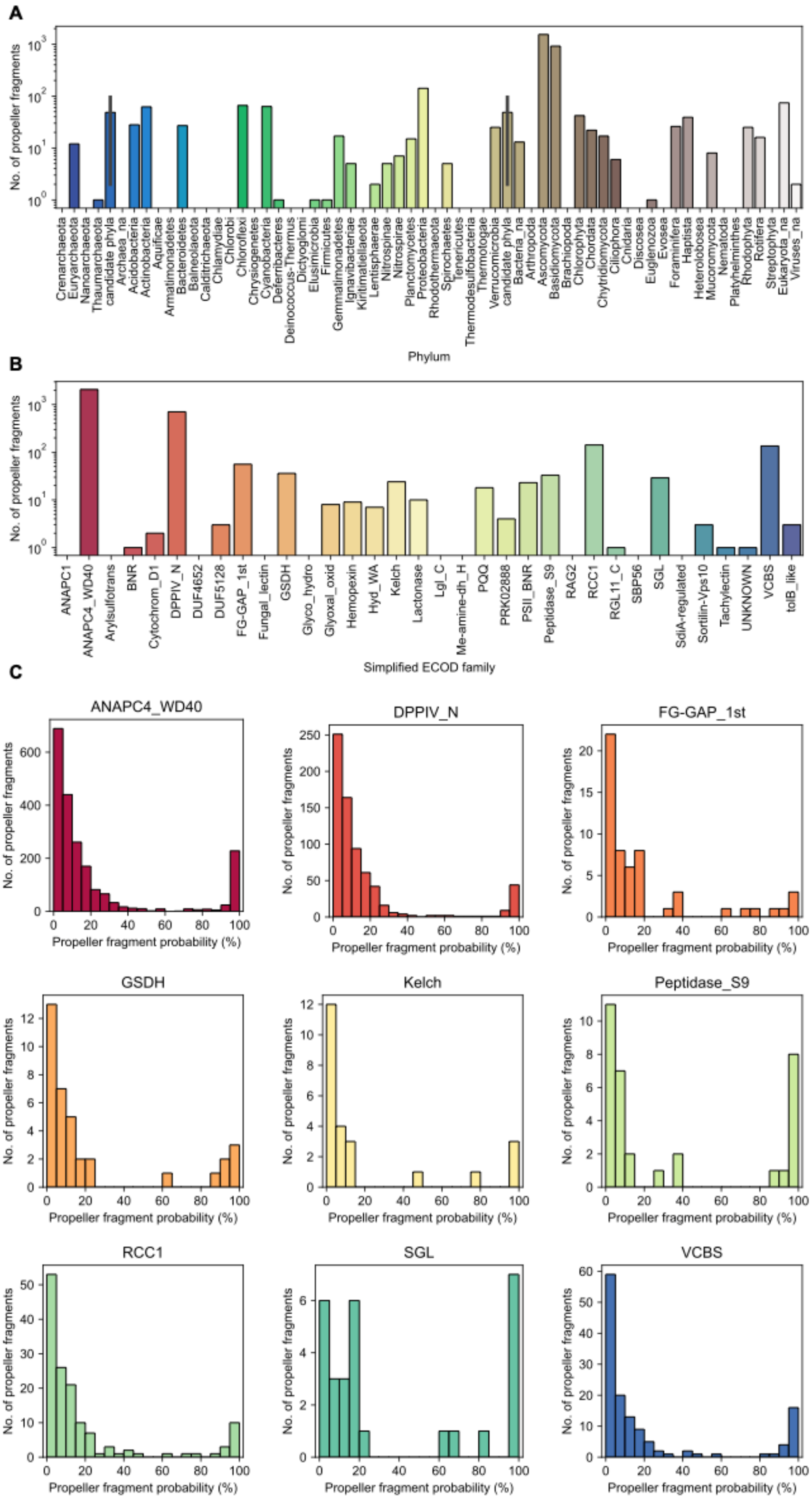

Figure S7. The overall distribution of putative β-propeller fragments. The number of fragments identified (a) per phyla and (b) highly repetitive β-propeller ECOD family. (c) Histograms of β-propeller fragments confidence (HHsearch probability) for the top nine families with the higher number of fragments.

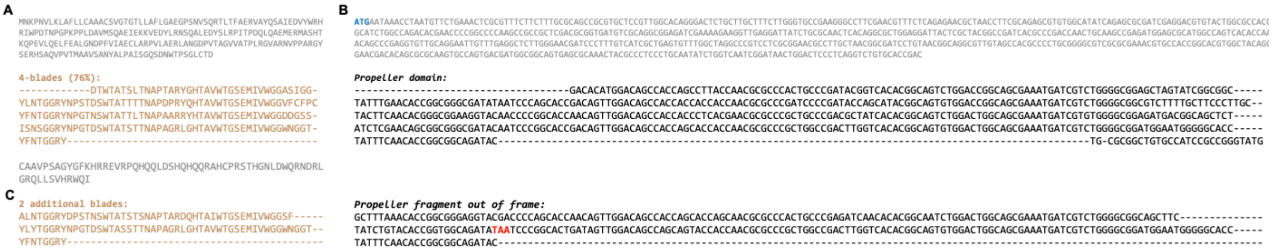

Figure S8. The β-propeller fragment in the neighbourhood of the globally repetitive β-propeller in hypothetical protein DMF28_08825 of an unknown Verrucomicrobia bacterium (PYL67592.1). (a) The protein sequence as deposited, highlighting the 4 highly repetitive blades. (b) The corresponding nucleotide sequence plus the 3’ genomic context. The start codon is highlighted in blue, and the in-frame stop codon in red. (c) The two additional blades hidden by a frame shift in the ORF that introduces the stop codon prematurely.
