# Supplementary material for: New β-Propellers Are Continuously Amplified From Single Blades in all Major Lineages of the β-Propeller Superfamily
Source: Front Mol Biosci. 2022 Jun 9;9:895496. doi: 10.3389/fmolb.2022.895496 (PMC9218822; doi:10.3389/fmolb.2022.895496)
Supplement: Supplementary file 8 [file DataSheet8.PDF]

A

MNKPNVLKLAFLLCAAACSVGTGTLLAFLGAEGPSNVSQRTLTFAERVAYQSAIEDVYWRH
RIWPDTNPGPKPPLDAVMSQAEIEKKVEDYLRNSQALEDYSLRPITPDQLQAEMERMASHT
KQPEVLQELFEALGNDPFVIAECLARPVLAERLANGDPVTAGVVATPLRGVARNVPPARGY
SERHSAQVPVTMAAVSANYALPAISGQSDNWTPSGLCTD

4-blades (76%):

```
------------DTWTATSLTNAPTARYGHTAVWTGSEMIVWGGASIGG-
YLNTGGRYNPSTDSWTATTTTNAPDPRYQHTAVWTGSEMIVWGGVFCFPC
YFNTGGRYNPGTNSWTATTLTNAPAARRYHTAVWTGSEMIVWGGDDGSS-
ISNSGGRYNPGTDSWTATSTTNAPAGRLGHTAVWTGSEMIVWGGWNGGT-
YFNTGGRY------------------------------------------
```

CAAVPSAGYGFKHRREVRPQHQQLDSHQHQQRAHCPRSTHGNLDWQRNDRL
GRQLLSVHRWQI

B

ATGAATAAACCTAATGTTCTGAAACTCGCGTTTCTTCTTTGCGCAGCCGCGTGCTCCGTTGGCACAGGGACTCTGCTTGCTTTCTTGGGTGCCGAAGGGCCTTCGAACGTTTCTCAGAGAACGCTAACCTTCGCAGAGCGTGTGGCATATCAGAGCGCGATCGAGGACGTGTACTGGCGCCACC
GCATCTGGCCAGACACGAACCCCGGCCCCAAGCCGCCGCTCGACGCGGTGATGTCGCAGGCGGAGATCGAAAAGAAGGTTGAGGATTATCTGCGCAACTCACAGGCGCTGGAGGATTACTCGCTACGGCCGATCACGCCCGACCAACTGCAAGCCGAGATGGAGCGCATGGCCAGTCACACCAA
ACAGCCCGAGGTGTTGCAGGAATTGTTTGAGGCTCTTGGGAACGATCCCTTTGTCATCGCTGAGTGTTTGGCTAGGCCCGTCCTCGCGGAACGCCTTGCTAACGGCGATCCTGTAACGGCAGGCGTTGTAGCCACGCCCCTGCGGGGCGTCGCGCGAAACGTGCCACCGGCACGTGGCTACAGC
GAACGACACAGCGCGCAAGTGCCAGTGACGATGGCGGCAGTGAGCGCAAACTACGCCCTCCCTGCAATATCTGGTCAATCGGATAACTGGACTCCCTCAGGTCTGTGCACCGAC

Propeller domain:

```
------------------------------------GACACATGGACAGCCACCAGCCTTACCAACGCGCCCACTGCCCGATACGGTCACACGGCAGTCTGGACCGGCAGCGAAATGATCGTCTGGGGCGGAGCTAGTATCGGCGGC-----
TATTTGAACACCGGCGGGCGATATAATCCCAGCACCGACAGTTGGACAGCCACCACCACCACCAACGCGCCCGATCCCCGATACCAGCATACGGCAGTGTGGACCGGCAGCGAAATGATCGTCTGGGGCGGCGTCTTTTGCTTCCCTTGC--
TACTTCAACACGGGCGGAAGGTACAACCCCGGCACCAACAGTTGGACAGCCACCACCCTCACGAACGCGCCCGCTGCCCGACGCTATCACACGGCAGTCTGGACTGGCAGCGAAATGATCGTGTGGGGCGGAGATGACGGCAGCTCT-----
ATCTCGAACAGCGGCGGGCGATACAATCCCGGCACCGACAGTTGGACAGCCACCAGCACCACCAACGCGCCCGCTGGCCGACTTGGTCACACGGCAGTGTGGACTGGCAGCGAAATGATCGTCTGGGGCGGATGGAATGGGGGCACC-----
TATTTCAACACCGGCGGCAGATAC---------------------------------------------------------------------------------------------------TG-CGCGGCTGTGCCATCCGCCGGGTATG
```

C

2 additional blades:

```
ALNTGGRYDPSTNSWTATSTSNAPTARDQHTAIWTGSEMIVWGGSF-----
YLYTGGRYNPGTDSWTASSTTNAPAGRLGHTAVWTGSEMIVWGGWNGGT--
YFNTGGRY-------------------------------------------
```

Propeller fragment out of frame:

```
GCTTTAAACACCGGCGGGAGGTACGACCCCAGCACCAACAGTTGGACAGCCACCAGCACCAGCAACGCGCCCACTGCCCGAGATCAACACACGGCAATCTGGACTGGCAGCGAAATGATCGTCTGGGGCGGCAGCTTC--------------
TATCTGTACACCGGTGGCAGATATAATCCCGGCACTGATAGTTGGACAGCCAGCAGTACCACCAACGCGCCCGCTGGCCGACTTGGTCACACGGCAGTGTGGACTGGCAGCGAAATGATCGTCTGGGGCGGATGGAATGGGGGCACC-----
TATTTCAACACCGGCGGCAGATAC--------------------------------------------------------------------------------------------------------------------------------
```
