# Supplementary material for: New β-Propellers Are Continuously Amplified From Single Blades in all Major Lineages of the β-Propeller Superfamily
Source: Front Mol Biosci. 2022 Jun 9;9:895496. doi: 10.3389/fmolb.2022.895496 (PMC9218822; doi:10.3389/fmolb.2022.895496)
Supplement: Supplementary file 4 [file DataSheet6.PDF]

A

(...)FSLTRRCYLEERSDWVLNEPLIDRDWSACLHTLE

47-blades (median sequence identity: 81%):

```
---SACLHTLEGHSYPVKSIAWSQDGSRLASASWDKTVRIWD
PTTGHCASTLKGHSSAVNSIAWSQDGNRLASASYDKTVMIWD
PTTDHCASTLKGHSGSVNSIAWTQDGSRLASASDDNTVRIWD
PTTGHCASTLKGHSSAVNSIAWSQDGNRLASASYDKTVMIWD
PTTDHCASTLKGHSGSVNSIAWTQDGSRLASASDDNTVRIWD
PTTGHCASTLKGHSSAVNSIAWSQDGNRLASASYDKTVMIWD
PTTDHCASTLKGHSGSVNSIAWTQDGSRLASASDDNTVRIWD
PTTGHCASTLKGHSSAVNSIAWSQDGNRLASASYDKTVMIWD
PTTDHCASTLKGHSGSVNSIAWTQDGSRLASASDDNTVRIWD
PTTGHCASTLKGHSSAVNSIAWSQDGNRLASASYDKTVMIWD
PTTDHCASTLKGHSGSVNSIAWTQDGSRLASASDDNTVRIWD
PTTGHCASTLKGHSSAVNSIAWSQDGNRLASASYDKTVMIWD
PTTDHCASTLKGHSGSVNSIAWTQDGSRLASASDDNTVRIWD
PTTGHCASTLKGHSSAVNSIAWSQDGNRLASASYDKTVMIWD
PTTDHCASTLKGHSGSVNSIAWTQDGSRLASASDDNTVRIWD
PTTGHCASTLKGHSSAVNSIAWSQDGNRLASASYDKTVMIWD
PTTDHCASTLKGHSGSVNSIAWTQDGSRLASASDDNTVRIWD
PTTGHCASTLKGHSSAVNSIAWSQDGNRLASASYDKTVMIWD
PTTDHCASTLKGHSGSVNSIAWTQDGSRLASASDDNTVRIWD
PTTGHCASTLKGHSSAVNSIAWSQDGNRLASASYDKTVMIWD
PTTDHCASTLKGHSGSVNSIAWTQDGSRLASASDDNTVRIWD
PTTGHCASTLKGHSSAVNSIAWSQDGNRLASASYDKTVMIWD
PTTDHCASTLKGHSGSVNSIAWTQDGSRLASASDDNTVRIWD
PTTGHCASTLKGHSSAVNSIAWSQDGNRLASASYDKTVMIWD
PTTDHCASTLKGHSGSVNSIAWTQDGSRLASASDDNTVRIWD
PTTGHCASTLKGHSSAVNSIAWSQDGNRLASASYDKTVMIWD
PTTDHCASTLKGHSGSVNSIAWTQDGSRLASASDDNTVRIWD
PTTGHCASTLKGHSSAVNSIAWSQDGNRLASASYDKTVMIWD
PTTDHCASTLKGHSGSVNSIAWTQDGSRLASASDDNTVRIWD
PTTGHCASTLKGHSSAVNSIAWSQDGNRLASASYDKTVMIWD
PTTDHCASTLKGHSGSVNSIAWTQDGSRLASASDDNTVRIWD
PTTGHCASTLKGHSSAVNSIAWSQDGNRLASASYDKTVMIWD
PTTDHCASTLKGHSGSVNSIAWTQDGSRLASASDDNTVRIWD
PTTGHCASTLKGHSSAVNSIAWSQDGNRLASASYDKTVMIWD
PTTDHCASTLKGHSGSVNSIAWTQDGSRLASASDDNTVRIWD
PTTGHCASTLKGHSSAVNSIAWSQDGNRLASASYDKTVMIWD
PTTDHCASTLKGHSGSVNSIAWTQDGSRLASASDDNTVRIWD
PTTGHCASTLKGHSSAVNSIAWSQDGNRLASASYDKTVMIWD
PTTDHCASTLKGHSGSVNSIAWTQDGSRLASASDDNTVRIWD
PTTGHCASTLKGHSSAVNSIAWSQDGNRLASASYDKTVMIWD
PTTDHCASTLKGHSGSVNSIAWTQDGSRLASASDDNTVRIWD
PTTGHCASTLKGHSSAVNSIAWSQDGNRLASASYDKTVMIWD
PTTDHCASTLKGHSGSVNSIAWTQDGSRLASASDDNTVRIWD
PTTGHCASTLKGHSSAVNSIAWSQDGNRLASASYDKTVMIWD
PTTDHCASTLKGHSGSVNSIAWTQDGSRLASASDDNTVRIWD
PTTGHCASTLKGHSSAVNSIAWSQDGNRLASASYDKTVMIWD
PTTDHCASTLKGHSGSVNSIAWTQDGS---------------
```

RHFERVCTSLMSHMVTFCWAVLSAPLQLTAWDKPNCTGDSAFTQFTNSVL
ARNISNALVSRSFKLSRALVDQEQLDISVTRNFTTWYSNKDQLSYKSSSC
TNFWQTYYAVNGSTACHNTPPFTCHRLWNNPGLPTQ

B

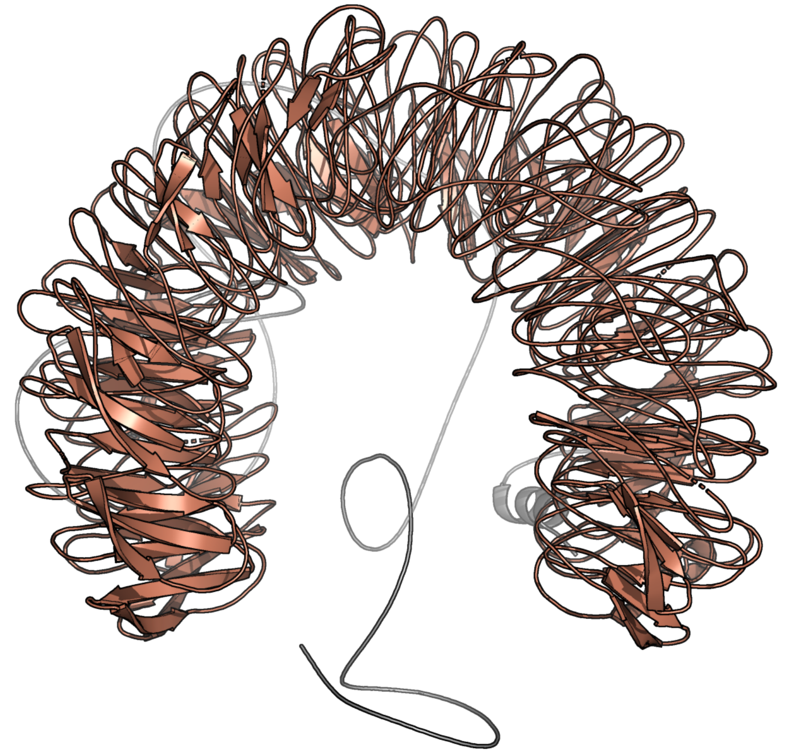

Model 1
pLDDT 66.3

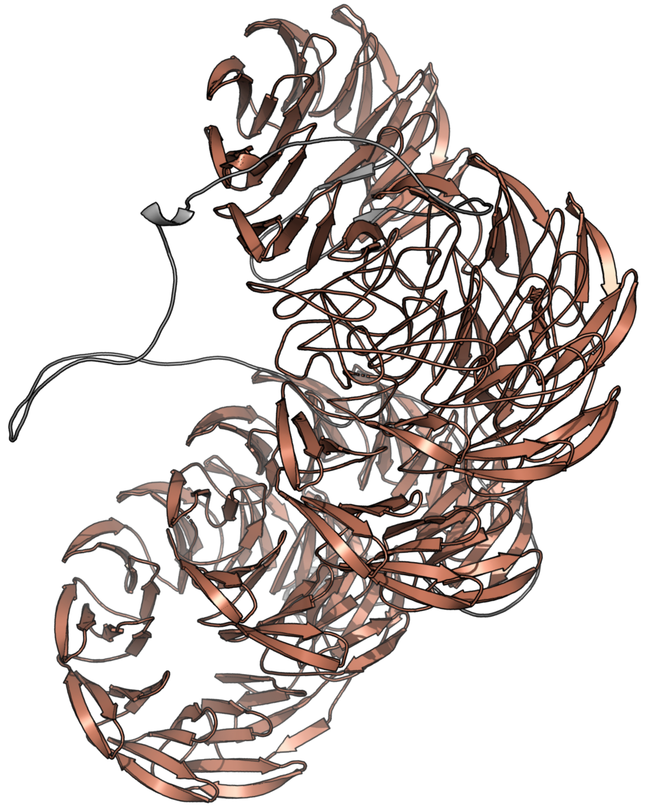

Model 2
pLDDT 60.6

C

```
---------AGTGCATGTCTTCATACCCTCGAGGGGCATAGCTATCCGGTCAAGTCGATTGCCTGGTCGCAGGATGGAAGCCGACTCGCATCAGCTTCCTGGGATAAAACCGTCAGGATTTGGGAT
CCGACTACCGGCCATTGCGCGTCAACCCTCAAGGGGCATAGCAGTGCGGTCAACTCGATTGCCTGGTCGCAAGATGGAAACCGACTCGCATCAGCGTCATATGATAAGACCGTCATGATCTGGGAT
CCGACCACCGACCATTGCGCGTCAACCCTCAAGGGGCATAGCGGTTCAGTCAACTCGATTGCCTGGACGCAAGACGGAAGCCGACTCGCATCAGCGTCAGATGATAACACCGTCAGGATTTGGGAT
CCGACTACCGGCCATTGCGCGTCAACCCTCAAGGGGCATAGCAGTGCGGTCAACTCGATTGCCTGGTCGCAAGATGGAAACCGACTCGCATCAGCGTCATATGATAAGACCGTCATGATCTGGGAT
CCGACCACCGACCATTGCGCGTCAACCCTCAAGGGGCATAGCGGTTCAGTCAACTCGATTGCCTGGACGCAAGACGGAAGCCGACTCGCATCAGCGTCAGATGATAACACCGTCAGGATTTGGGAT
CCGACTACCGGCCATTGCGCGTCAACCCTCAAGGGGCATAGCAGTGCGGTCAACTCGATTGCCTGGTCGCAAGATGGAAACCGACTCGCATCAGCGTCATATGATAAGACCGTCATGATCTGGGAT
CCGACCACCGACCATTGCGCGTCAACCCTCAAGGGGCATAGCGGTTCAGTCAACTCGATTGCCTGGACGCAAGACGGAAGCCGACTCGCATCAGCGTCAGATGATAACACCGTCAGGATTTGGGAT
CCGACTACCGGCCATTGCGCGTCAACCCTCAAGGGGCATAGCAGTGCGGTCAACTCGATTGCCTGGTCGCAAGATGGAAACCGACTCGCATCAGCGTCATATGATAAGACCGTCATGATCTGGGAT
CCGACCACCGACCATTGCGCGTCAACCCTCAAGGGGCATAGCGGTTCAGTCAACTCGATTGCCTGGACGCAAGACGGAAGCCGACTCGCATCAGCGTCAGATGATAACACCGTCAGGATTTGGGAT
CCGACTACCGGCCATTGCGCGTCAACCCTCAAGGGGCATAGCAGTGCGGTCAACTCGATTGCCTGGTCGCAAGATGGAAACCGACTCGCATCAGCGTCATATGATAAGACCGTCATGATCTGGGAT
CCGACCACCGACCATTGCGCGTCAACCCTCAAGGGGCATAGCGGTTCAGTCAACTCGATTGCCTGGACGCAAGACGGAAGCCGACTCGCATCAGCGTCAGATGATAACACCGTCAGGATTTGGGAT
CCGACTACCGGCCATTGCGCGTCAACCCTCAAGGGGCATAGCAGTGCGGTCAACTCGATTGCCTGGTCGCAAGATGGAAACCGACTCGCATCAGCGTCATATGATAAGACCGTCATGATCTGGGAT
CCGACCACCGACCATTGCGCGTCAACCCTCAAGGGGCATAGCGGTTCAGTCAACTCGATTGCCTGGACGCAAGACGGAAGCCGACTCGCATCAGCGTCAGATGATAACACCGTCAGGATTTGGGAT
CCGACTACCGGCCATTGCGCGTCAACCCTCAAGGGGCATAGCAGTGCGGTCAACTCGATTGCCTGGTCGCAAGATGGAAACCGACTCGCATCAGCGTCATATGATAAGACCGTCATGATCTGGGAT
CCGACCACCGACCATTGCGCGTCAACCCTCAAGGGGCATAGCGGTTCAGTCAACTCGATTGCCTGGACGCAAGACGGAAGCCGACTCGCATCAGCGTCAGATGATAACACCGTCAGGATTTGGGAT
CCGACTACCGGCCATTGCGCGTCAACCCTCAAGGGGCATAGCAGTGCGGTCAACTCGATTGCCTGGTCGCAAGATGGAAACCGACTCGCATCAGCGTCATATGATAAGACCGTCATGATCTGGGAT
CCGACCACCGACCATTGCGCGTCAACCCTCAAGGGGCATAGCGGTTCAGTCAACTCGATTGCCTGGACGCAAGACGGAAGCCGACTCGCATCAGCGTCAGATGATAACACCGTCAGGATTTGGGAT
CCGACTACCGGCCATTGCGCGTCAACCCTCAAGGGGCATAGCAGTGCGGTCAACTCGATTGCCTGGTCGCAAGATGGAAACCGACTCGCATCAGCGTCATATGATAAGACCGTCATGATCTGGGAT
CCGACCACCGACCATTGCGCGTCAACCCTCAAGGGGCATAGCGGTTCAGTCAACTCGATTGCCTGGACGCAAGACGGAAGCCGACTCGCATCAGCGTCAGATGATAACACCGTCAGGATTTGGGAT
CCGACTACCGGCCATTGCGCGTCAACCCTCAAGGGGCATAGCAGTGCGGTCAACTCGATTGCCTGGTCGCAAGATGGAAACCGACTCGCATCAGCGTCATATGATAAGACCGTCATGATCTGGGAT
CCGACCACCGACCATTGCGCGTCAACCCTCAAGGGGCATAGCGGTTCAGTCAACTCGATTGCCTGGACGCAAGACGGAAGCCGACTCGCATCAGCGTCAGATGATAACACCGTCAGGATTTGGGAT
CCGACTACCGGCCATTGCGCGTCAACCCTCAAGGGGCATAGCAGTGCGGTCAACTCGATTGCCTGGTCGCAAGATGGAAACCGACTCGCATCAGCGTCATATGATAAGACCGTCATGATCTGGGAT
CCGACCACCGACCATTGCGCGTCAACCCTCAAGGGGCATAGCGGTTCAGTCAACTCGATTGCCTGGACGCAAGACGGAAGCCGACTCGCATCAGCGTCAGATGATAACACCGTCAGGATTTGGGAT
CCGACTACCGGCCATTGCGCGTCAACCCTCAAGGGGCATAGCAGTGCGGTCAACTCGATTGCCTGGTCGCAAGATGGAAACCGACTCGCATCAGCGTCATATGATAAGACCGTCATGATCTGGGAT
CCGACCACCGACCATTGCGCGTCAACCCTCAAGGGGCATAGCGGTTCAGTCAACTCGATTGCCTGGACGCAAGACGGAAGCCGACTCGCATCAGCGTCAGATGATAACACCGTCAGGATTTGGGAT
CCGACTACCGGCCATTGCGCGTCAACCCTCAAGGGGCATAGCAGTGCGGTCAACTCGATTGCCTGGTCGCAAGATGGAAACCGACTCGCATCAGCGTCATATGATAAGACCGTCATGATCTGGGAT
CCGACCACCGACCATTGCGCGTCAACCCTCAAGGGGCATAGCGGTTCAGTCAACTCGATTGCCTGGACGCAAGACGGAAGCCGACTCGCATCAGCGTCAGATGATAACACCGTCAGGATTTGGGAT
CCGACTACCGGCCATTGCGCGTCAACCCTCAAGGGGCATAGCAGTGCGGTCAACTCGATTGCCTGGTCGCAAGATGGAAACCGACTCGCATCAGCGTCATATGATAAGACCGTCATGATCTGGGAT
CCGACCACCGACCATTGCGCGTCAACCCTCAAGGGGCATAGCGGTTCAGTCAACTCGATTGCCTGGACGCAAGACGGAAGCCGACTCGCATCAGCGTCAGATGATAACACCGTCAGGATTTGGGAT
CCGACTACCGGCCATTGCGCGTCAACCCTCAAGGGGCATAGCAGTGCGGTCAACTCGATTGCCTGGTCGCAAGATGGAAACCGACTCGCATCAGCGTCATATGATAAGACCGTCATGATCTGGGAT
CCGACCACCGACCATTGCGCGTCAACCCTCAAGGGGCATAGCGGTTCAGTCAACTCGATTGCCTGGACGCAAGACGGAAGCCGACTCGCATCAGCGTCAGATGATAACACCGTCAGGATTTGGGAT
CCGACTACCGGCCATTGCGCGTCAACCCTCAAGGGGCATAGCAGTGCGGTCAACTCGATTGCCTGGTCGCAAGATGGAAACCGACTCGCATCAGCGTCATATGATAAGACCGTCATGATCTGGGAT
CCGACCACCGACCATTGCGCGTCAACCCTCAAGGGGCATAGCGGTTCAGTCAACTCGATTGCCTGGACGCAAGACGGAAGCCGACTCGCATCAGCGTCAGATGATAACACCGTCAGGATTTGGGAT
CCGACTACCGGCCATTGCGCGTCAACCCTCAAGGGGCATAGCAGTGCGGTCAACTCGATTGCCTGGTCGCAAGATGGAAACCGACTCGCATCAGCGTCATATGATAAGACCGTCATGATCTGGGAT
CCGACCACCGACCATTGCGCGTCAACCCTCAAGGGGCATAGCGGTTCAGTCAACTCGATTGCCTGGACGCAAGACGGAAGCCGACTCGCATCAGCGTCAGATGATAACACCGTCAGGATTTGGGAT
CCGACTACCGGCCATTGCGCGTCAACCCTCAAGGGGCATAGCAGTGCGGTCAACTCGATTGCCTGGTCGCAAGATGGAAACCGACTCGCATCAGCGTCATATGATAAGACCGTCATGATCTGGGAT
CCGACCACCGACCATTGCGCGTCAACCCTCAAGGGGCATAGCGGTTCAGTCAACTCGATTGCCTGGACGCAAGACGGAAGCCGACTCGCATCAGCGTCAGATGATAACACCGTCAGGATTTGGGAT
CCGACTACCGGCCATTGCGCGTCAACCCTCAAGGGGCATAGCAGTGCGGTCAACTCGATTGCCTGGTCGCAAGATGGAAACCGACTCGCATCAGCGTCATATGATAAGACCGTCATGATCTGGGAT
CCGACCACCGACCATTGCGCGTCAACCCTCAAGGGGCATAGCGGTTCAGTCAACTCGATTGCCTGGACGCAAGACGGAAGCCGACTCGCATCAGCGTCAGATGATAACACCGTCAGGATTTGGGAT
CCGACTACCGGCCATTGCGCGTCAACCCTCAAGGGGCATAGCAGTGCGGTCAACTCGATTGCCTGGTCGCAAGATGGAAACCGACTCGCATCAGCGTCATATGATAAGACCGTCATGATCTGGGAT
CCGACCACCGACCATTGCGCGTCAACCCTCAAGGGGCATAGCGGTTCAGTCAACTCGATTGCCTGGACGCAAGACGGAAGCCGACTCGCATCAGCGTCAGATGATAACACCGTCAGGATTTGGGAT
CCGACTACCGGCCATTGCGCGTCAACCCTCAAGGGGCATAGCAGTGCGGTCAACTCGATTGCCTGGTCGCAAGATGGAAACCGACTCGCATCAGCGTCATATGATAAGACCGTCATGATCTGGGAT
CCGACCACCGACCATTGCGCGTCAACCCTCAAGGGGCATAGCGGTTCAGTCAACTCGATTGCCTGGACGCAAGACGGAAGCCGACTCGCATCAGCGTCAGATGATAACACCGTCAGGATTTGGGAT
CCGACTACCGGCCATTGCGCGTCAACCCTCAAGGGGCATAGCAGTGCGGTCAACTCGATTGCCTGGTCGCAAGATGGAAACCGACTCGCATCAGCGTCATATGATAAGACCGTCATGATCTGGGAT
CCGACCACCGACCATTGCGCGTCAACCCTCAAGGGGCATAGCGGTTCAGTCAACTCGATTGCCTGGACGCAAGACGGAAGCCGACTCGCATCAGCGTCAGATGATAACACCGTCAGGATTTGGGAT
CCGACTACCGGCCATTGCGCGTCAACCCTCAAGGGGCATAGCAGTGCGGTCAACTCGATTGCCTGGTCGCAAGATGGAAACCGACTCGCATCAGCGTCATATGATAAGACCGTCATGATCTGGGAT
CCGACCACCGACCATTGCGCGTCAACCCTCAAGGGGCATAGCGGTTCAGTCAACTCGATTGCCTGGACGCAAGACGGAAGC---------------------------------------------
```
